# Microneurolysis and decompression of long thoracic nerve injury are effective in reversing scapular winging: Long-term results in 50 cases

**DOI:** 10.1186/1471-2474-8-25

**Published:** 2007-03-07

**Authors:** Rahul K Nath, Andrew B Lyons, Gabriel Bietz

**Affiliations:** 1 Director, Texas Nerve and Paralysis Institute, Houston, Texas 77030, USA; 2 Research Associate, Texas Nerve and Paralysis Institute, Houston, Texas 77030, USA

## Abstract

**Background:**

Long thoracic nerve injury leading to scapular winging is common, often caused by closed trauma through compression, stretching, traction, direct extrinsic force, penetrating injury, or neuritides such as Parsonage-Turner syndrome. We undertook the largest series of long thoracic nerve decompression and neurolysis yet reported to demonstrate the usefulness of long thoracic nerve decompression.

**Methods:**

Winging was bilateral in 3 of the 47 patients (26 male, 21 female), yielding a total of 50 procedures. The mean age of the patients was 33.4 years, ranging from 24–57. Causation included heavy weight-lifting (31 patients), repetitive throwing (5 patients), deep massage (2 patients), repetitive overhead movement (1 patient), direct trauma (1 patient), motor bike accident (1 patient), and idiopathic causes (9 patients). Decompression and microneurolysis of the long thoracic nerve were performed in the supraclavicular space. Follow-up (average of 25.7 months) consisted of physical examination and phone conversations. The degree of winging was measured by the operating surgeon (RKN). Patients also answered questions covering 11 quality-of-life facets spanning four domains of the World Health Organization Quality of Life questionnaire.

**Results:**

Thoracic nerve decompression and neurolysis improved scapular winging in 49 (98%) of the 50 cases, producing "good" or "excellent" results in 46 cases (92%). At least some improvement occurred in 98% of cases that were less than 10 years old. Pain reduction through surgery was good or excellent in 43 (86%) cases. Shoulder instability affected 21 patients preoperatively and persisted in 5 of these patients after surgery, even in the 5 patients with persistent instability who experienced some relief from the winging itself.

**Conclusion:**

Surgical decompression and neurolysis of the long thoracic nerve significantly improve scapular winging in appropriate patients, for whom these techniques should be considered a primary modality of functional restoration.

## Background

Winging of the scapula due to long thoracic nerve palsy is a common diagnosis [1-10] and a significant functional problem, not simply an aesthetic issue. Secondary pain and spasm result from muscle imbalances and tendonitis around the shoulder joint caused by muscular activity that compensates for impaired shoulder stability. Winging also leads to adhesive capsulitis, subacromial impingement, and brachial plexus radiculitis [5]. Traditional management has relied on conservative therapy [2,3,11-14] and pectoralis tendon transfer for refractory cases [4,5,13-15]. A less common approach has been scapulothoracic arthrodesis [16]. Most patients probably never undergo surgery and hence live with chronic shoulder instability and pain.

The unique anatomy of the long thoracic nerve predisposes it to injury, which often causes scapular winging. Compared to the relatively robust adjacent nerves of the brachial plexus, the long thoracic nerve is small in diameter, appears fragile, and has much less (and much more translucent) connective tissue. The lengthy course of the nerve also increases chances of injury. Surgical dissection in the axilla can directly injure the nerve in the infraclavicular region in as many as 30% of procedures [17].

The anatomic feature usually associated with injury is the long thoracic nerve's course through the fibers of the middle scalene muscle in the supraclavicular region [18-20]. In several of our patients, nerve injury appeared to have occurred during exercise when the contractions of the middle scalene muscle directly compressed the long thoracic nerve. Another injury category involves an extrinsic crushing of the nerve in the vicinity of the middle scalene muscle, a possible secondary source of injury. Most of our patients relate a history of strenuous upper-extremity activity or heavy weight-lifting. Deep massage treatments directly compressed the supraclavicular fossa in two patients, with associated pain and paresthesia during treatment.

Scapular winging can be treated by directly addressing the nerve injury. Nerve transfer surgery can be used to restore long thoracic nerve function [21,22]. A simpler surgical approach for continuous nerves with evidence of compression in the scalene muscles is decompression and microneurolysis. Disa and coworkers [19] reported successful neurolysis of the long thoracic nerve in four patients with winging caused by stretching or trauma involving the middle scalene [18]. This study reports 50 cases in which decompression and neurolysis of the long thoracic nerve where it traverses the middle scalene improved scapular winging.

## Methods

Forty-seven consecutive patients consisting of 26 males and 21 females with a mean age of 33.4 years (24–57 years) underwent surgery after evaluation for a winging scapula. All patients gave informed consent to participate in this study. The investigation was carried out in accordance with the Declaration of Helsinki. Winging was bilateral and symptomatic in three patients; three others had clear but asymptomatic winging of the contralateral side. The total number of operations was 50. The most common symptoms were initial discomfort and spasm of the affected shoulder girdle muscles with shoulder instability and winging of the scapula. Thirty-one patients (62%) could not abduct and flex the shoulder beyond 90 degrees. Of the 50 cases, 32 were right-sided, 18 left-sided nerve injuries. Most patients were right-hand dominant.

Thirty-one (62%) had a history of weight-lifting. Winging followed rigorous, extended throwing exercises in five patients (10%). In two patients (4%), winging immediately followed deep massage in the area of the supraclavicular fossa, and one patient (2%) was a postal worker who had performed repetitive overhead movements for several years. One patient (2%) had been hit by a ladder at work and gave a detailed history of direct trauma to the supraclavicular area. A motorbike accident caused winging in one patient (2%) when his arm and shoulder were jerked forward sharply while he gripped the handlebars during a fall. Winging was idiopathic in nine patients (18%) and considered to be related to Parsonage-Turner syndrome. It is our belief, however, that many times Parsonage-Turner Syndrome is misdiagnosed because of associated atypical symptoms that can often be related to previous strenuous physical activity.

## Patient Evaluation

The senior author, an experienced brachial plexus surgeon, performed all surgery. Each surgical procedure followed the same protocol (defined below). The same author (ABL) performed follow-up evaluation (average, 25.7 months).

Physical examination uniformly revealed medial deviation of the inferior angle of the scapula and prominent winging of the medial border of the scapula with backward pressure on the shoulder during forward protraction. The scapula was also elevated superiorly. Patients experienced significant discomfort and feelings of shoulder instability with overhead arm and shoulder movements: 31 (62%) of 50 patients could not flex or abduct the shoulder beyond 90 degrees.

No grading systems for the serratus anterior muscle have been established, so we quantified the degree of winging by estimating the angle between the plane of the scapula and the posterior chest wall. British Motor Grading (BMG), a grading system outlined by the Medical Research Council for semi-quantitative assessment of muscle strength, applied to upper-trunk examination [23]. Muscle function is graded from M0 to M5 with M0 being complete absence of contraction and M5 being normal function.

Physical examination revealed weakness of the deltoid, spinati, and biceps muscles (BMG, M3-M4) in 38 cases, a finding consistent with concurrent injury to the upper trunk of the brachial plexus.

Electromyography of the brachial plexus and long thoracic nerve distribution was performed prior to physical examination, in all cases. Serratus anterior abnormalities were present in 44 (88%) patients; subtle, transient abnormalities of the serratus examination were detected in 3 (6%). Abnormal EMG results all described neuropraxic injury with no loss of axonal continuity. Presurgery MRI was performed in 12 patients and yielded normal results except in 9 cases showing atrophy of the serratus anterior muscle. Intraoperative nerve action potentials were not tested in all cases, which may have potentially shown regeneration after a 3 month interval despite a lack of clinical recovery.

All 47 patients had undergone regular physical therapy prior to surgery. Eight patients who had experienced symptoms for longer than 7 years described possible minor improvement with conservative management, but all felt that winging constrained the scope and intensity of their physical activity.

To qualify for surgery, the injury generally had to have occurred at least 3 months before surgery (there were four exceptions). The average time interval between the time of injury and surgical treatment was 8 months. Progressive improvement with physical therapy was another disqualifying factor; slowly progressive symptoms or failed conservative management were qualifying factors. Another consideration was a history that strongly suggested injury to the long thoracic nerve in the region of the middle scalene muscle. Scapular winging and proximal extremity weakness after the lifting of heavy weights and aggressive throwing motions do support the theory of middle and anterior scalene compression of contained nerves. Another important cause was thought to be direct extrinsic pressure to the relatively superficial long thoracic nerve and the upper plexus in the supraclavicular area. Stretching and axial traction of these nerve elements by various mechanisms was considered significant. Abnormal EMG results were considered to confirm the presence of severe injury, but the lack of such abnormalities was not considered a contraindication to surgery when clinical evidence strongly supported severe injury.

Surgical candidates participated in a thorough discussion of the risks and potential benefits. Discussed options were continuing conservative management, pectoralis tendon transfer, or scapulothoracic fusion. Patients understood that we considered an excellent outcome less likely if winging was more than 10 years old, given our knowledge of denervated skeletal muscle, including the serratus anterior.

Follow-up consisted of serial physical examination (same examiner) and long-term postoperative evaluation through phone conversations (WHO WHOQOL-100 field trial questionnaire) in which a 1–5 scaled was used to assess changes in 11 facets of four quality-of-life domains. The Wilcoxon signed ranks test was used for statistical analysis.

## Surgical Technique

Patients were placed in the lawn-chair position, with a shoulder roll. The head and neck were abducted away from the side of surgery. The entire supraclavicular area was prepared and draped with a thyroid sheet. The skin incision was created two fingerbreadths posterior and parallel to the clavicle. The incision was sinusoidal and extended 6–8 cm lateral to the palpated lateral clavicular border of the sternocleidomastoid muscle. Dissection proceeded through the platysma muscle while carefully protecting the underlying supraclavicular nerves. Resection of the omohyoid muscle allowed access to the scalene fat pad and removal of a potential compressive structure of the brachial plexus. Inferior to superior elevation of the scalene fat pad revealed the upper brachial plexus. Identification of the suprascapular branch of the upper trunk required great care: this branch often traverses the middle layers of the scalene fat pad, producing a potential site of iatrogenic injury.

The next stage involved exploration of the upper trunk and its trifurcation into the anterior and posterior divisions and the suprascapular nerve. Epineurial scarring was typical at this point, and microsurgical instruments and technique were used for external neurolysis. Simultaneous anterior scalene resection was generally partial, releasing only the most superficial fibers compressing the upper trunk. Thus, resection typically involved 15%–20% of the thickness of the anterior scalene muscle.

The long thoracic nerve was then exposed laterally and posteriorly to the upper trunk. The long thoracic nerve and its tributaries are delicate here, no more than 2–3 mm in diameter. Passage of the nerve through the thick middle scalene muscle and the nerve's slightness compared to the serratus anterior muscle may predispose the neuromuscular unit to dysfunction [18].

Internal and external neurolysis of the isolated nerve was performed with microsurgical instruments and the operating microscope in light of the nerve's delicate nature and to reduce surgical scar formation. The long thoracic nerve has several fascicles, visible under magnification and previously dissected and described anatomically by Zancolli and Cozzi [24]. Internal neurolysis was performed in all cases as a standard approach to repair. The middle scalene was resected enough to decompress the long thoracic nerve and its branches as they traverse and exit the muscle. A demarcated area of compression within the nerve was apparent in 47 (94%) of the 50 procedures, more so toward the point of exit from the muscle. The site of compression showed narrowing and surface neovascularization of the epineurium. One case required complete resection of the middle scalene; the other 49 cases required partial release, which was enough to expose the long thoracic nerve and remove the circumferential muscle fibers.

Prior to closure, the superior-most and inferior margins of the surgical wound were examined via direct vision and sharp dissection to identify and release compressive fascial bands that might compress the upper trunk and long thoracic nerve.

Closure involved reconstruction of the platysma and two skin layers with no drains. Immediate postoperative management consisted of active range of motion at the shoulder and neck. The goal for the third postoperative day was full range of motion at or beyond preoperative levels.

## Results

As noted earlier, our series of 50 long thoracic nerve decompression and neurolysis procedures is the largest such series in the literature. Forty-four (88%) of these procedures significantly improved scapular winging within 1 day to 3 months. Winging improved in 98% of patients in whom it had been present for less than 10 years. Subjectively speaking, decompression and neurolysis appeared to improve contractions of the serratus anterior. Recovery, as assessed through British Motor Grade muscle strength analysis and estimating the decrease in the angle between the plane of the scapula and the posterior chest wall, occurred within 24 hours in 50% of cases.

Half of the patients experienced pain, which was improved in 73% of cases. All 31 patients who had shoulder instability that interfered with abduction and shoulder flexing beyond 90 degrees experienced relief following surgery.

In 8 (9%) of 50 extremities, patients reported a 2 cm^2 ^swelling at the area of incision between 3 and 6 weeks after surgery, which in all cases resolved spontaneously within 1 week and may have represented an unusually late-appearing seroma. The prevalence of swelling in these cases occurred equally bilaterally.

All 47 patients completed the WHOQOL-100 questionnaire. The average quality-of-life rating was 3.4 ("excellent") for all 11 facets. Except for three patients who considered their appearance, pain level, or sleep to have worsened slightly, all responses affirmed improvement. These three patients included one, a 32 year old male, with winging persisting over 10 years. The other two correspond to one patient, a 28 year old male with a severe case of bilateral winging.

All 11 QOL categories achieved significance (p <0.001 – see Table 1). The average response to surgery was 3.6 (treatment within 2 years of the onset of winging) and 1.84 (treatment after 8 years from onset). No preoperative survey was administered because of the nature of the WHOQOL-100 questionnaire as a historical survey.

**Figure 1:**
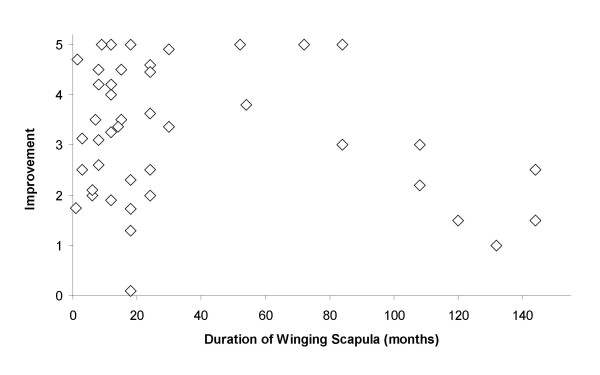
Quality of life improvement in 50 scapular winging instances.

Poorer outcomes were associated with delays longer than 8 years between onset of winging to surgery (Figure 1). No infections or other complications occurred. One patient who had been injured while playing softball experienced partial recurrence 6 months after initially showing improvement, but this was due to premature resumption of competitive softball pitching. The recurrence had not resolved 6 months after beginning, and the patient is being considered for a pectoralis tendon transfer.

## Discussion

Scapular winging is an important cause of functional disability. The morbidity associated with long thoracic nerve dysfunction is underappreciated and under treated. Ferry [25] argues that conservative treatment is an option based on inadequate data; we likewise favor a more aggressive approach involving surgery. Ten of our patients had significant functional deficits due to winging that had been present for more than 6 years; all 10 responded to surgery, often (70% of cases) within 1 week of surgery. The average time to improvement was 5 days (range 1 day to 3 months).

Our findings support nerve surgery as a treatment option in specific cases of supraclavicular injury to the long thoracic nerve and diminish the possibility that improvement was spontaneous. Risk factors for supraclavicular nerve injury include vigorous athletic maneuvers with the affected extremity, lifting heavy weights, and direct external pressure (as in deep massage). Injury to the upper trunk of the brachial plexus is also associated with the proposed stretch or compression mechanisms of injury. Upper-trunk disease reduces the reliability of shoulder examination because deltoid and spinati strength are reduced by long- standing scapular instability. However, all of our patients showed direct evidence of upper-trunk injury (BMG biceps score of M3 or M4).

The relatively delicate structure of the long thoracic nerve is contrasted with the densely composed upper trunk and predicts the consequences of trauma to each element: given similarly applied forces, the upper trunk shows less dysfunction than the long thoracic nerve. This explains why electrophysiologic examination of the upper trunk-supplied muscles of the affected extremity often reveals no clear abnormalities, the upper trunk injury being relatively minor [19,26]. EMG of the serratus anterior, however, often reveals greater dysfunction than in the other muscles. Unfortunately, electrodiagnostic studies of the long thoracic nerve can be unreliable indicators of injury severity[27]. The tendency toward normal results with serratus anterior testing in our population may then be ascribed to inadvertent testing of the latissimus dorsi, teres major, or other unaffected chest wall muscles, which can be difficult to isolate [28]. The three main portions of the serratus anterior are preferentially innervated by branches from different roots of the long thoracic nerve [29], and both the stimulation point and measurement point in relationship to the nerve constriction site could all affect the EMG results [28]. The fact that the long thoracic nerve was in continuity in all 50 cases might also have hampered EMG-based attempts to uncover subtle (but functionally significant) denervation abnormalities.

The observed rapid recovery of half of the patients might indicate a less severe nerve injury (no focal demyelination) that remains functionally debilitating. It is unlikely that the fast recovery is due to an ischemic mechanism, since muscle atrophy and denervation would be expected in cases persisting several years. Such injury may be classified as a Sunderland grade 0.5, wherein the myelin of the injured nerves is apparently intact and a different mechanism causes loss of function [30,31]. One possibility is the concept of "silent synapses" – inhibited acetylcholine release at the motor end plate due to blocked axonal flow caused by mechanical forces. Transport is sufficient to sustain nerve health, but not to stimulate muscle contraction. By surgically removing the source of nerve constriction, normal muscle function could quickly resume [30].

This study expands the database of nerve-based management of scapular winging caused by supraclavicular long thoracic nerve injury. Muscle- and tendon-based procedures should remain important tools in cases of refractory, symptomatic scapular winging, but the effectiveness and low morbidity of long thoracic nerve decompression appear to justify its becoming the initial treatment of choice in appropriate cases, for which we suggest the following paradigm:

1. Clinical evaluation with particular attention to the cause of injury or any associated events. Patients with symptomatic scapular winging related to injury localized at the long thoracic nerve near the middle scalene are candidates for nerve surgery.

2. Direct physical examination, with particular attention to scapular movements and strength. Strength of the serratus anterior muscle can be measured in terms of maximal protrusion at the inferior scapular angle (normal is 0 cm; >5 cm signifies extreme loss of function).

3. Electrical studies to detect loss of nerve continuity, which could indicate the need for nerve grafting or transfer. The lack of abnormal findings with electrical testing should not obviate surgery when clinical findings suggest surgical intervention.

4. Observation of the following guidelines for determining surgical candidacy and time since onset of injury:

• <7 years, candidate for nerve surgery in the absence of other contraindications such as established loss of nerve continuity for > 18 months

• 7–10 years, relative candidate (good outcomes are less predictable)

• >10 years, tendon transfer becomes the primary treatment option (nerve surgery is a secondary option)

Pain and inflammation do not automatically improve even with successful flattening of the scapula, and they improve unpredictably in our experience. We find that patients are not always happy with the results of surgery when the primary presenting symptom is established pain, even when the winging is reversed. These cases, however, represent a minority of all patient experiences.

Postoperative management is generally restricted to gentle range-of-motion (ROM) therapy and electrical stimulation. The protocol for patients with longstanding winging is daily stretching for up to 1 year following surgery, after which strengthening work can begin. Patients with winging for less than 2 years begin strengthening after 3 months of ROM therapy, which helps prevent and treat adhesive capsulitis at various shoulder girdle joints.

## Conclusion

Winging of the scapula is an important but underappreciated public health problem. Many sufferers live with pain and ongoing functional deficits. Peripheral nerve surgery is becoming increasingly important in managing scapular winging related to the long thoracic nerve, often treating the cause of the problem rather than the result of the injury. In many cases, the effectiveness and low morbidity of nerve surgery make it the treatment of choice. Ongoing research into surgical and conservative management will further improve the management of scapular winging.

## Competing interests

The author(s) declare that they have no competing interests.

## Authors' contributions

RKN Performed surgery, conceived of the study, analyzed data, and wrote the paper. ABL and GB both collected and analyzed data. All authors read and approved the final manuscript.

## Figures and Tables

**Table 1:** Quality of life questionnaire statistics

**Facet**	**Z-score**	**p value**
Energy	-3.845	< 0.001
Pain	-3.741	< 0.001
Sleep	-3.736	< 0.001
Appearance	-3.837	< 0.001
Feelings	-3.838	< 0.001
Range of Motion	-3.745	< 0.001
Activities of daily living	-3.839	< 0.001
Medications	-3.843	< 0.001
Work	-3.640	< 0.001
Relationships	-3.744	< 0.001
Overall	-3.736	< 0.001
